# SILVA tree viewer: interactive web browsing of the SILVA phylogenetic guide trees

**DOI:** 10.1186/s12859-017-1841-3

**Published:** 2017-09-30

**Authors:** Alan Beccati, Jan Gerken, Christian Quast, Pelin Yilmaz, Frank Oliver Glöckner

**Affiliations:** 1 Department of Life Sciences & Chemistry, Jacobs University gGmbH, Bremen, Germany; 2 Microbial Genomics and Bioinformatics Research Group, Max Planck Institute for Marine Microbiology, Bremen, Germany

## Abstract

**Background:**

Phylogenetic trees are an important tool to study the evolutionary relationships among organisms. The huge amount of available taxa poses difficulties in their interactive visualization. This hampers the interaction with the users to provide feedback for the further improvement of the taxonomic framework.

**Results:**

The SILVA Tree Viewer is a web application designed for visualizing large phylogenetic trees without requiring the download of any software tool or data files. The SILVA Tree Viewer is based on Web Geographic Information Systems (Web-GIS) technology with a PostgreSQL backend. It enables zoom and pan functionalities similar to Google Maps. The SILVA Tree Viewer enables access to two phylogenetic (guide) trees provided by the SILVA database: the SSU Ref NR99 inferred from high-quality, full-length small subunit sequences, clustered at 99% sequence identity and the LSU Ref inferred from high-quality, full-length large subunit sequences.

**Conclusions:**

The Tree Viewer provides tree navigation, search and browse tools as well as an interactive feedback system to collect any kinds of requests ranging from taxonomy to data curation and improving the tool itself.

## Background

Reconstructing phylogenetic trees is an important method for studying the evolutionary relations among organisms. In molecular phylogeny, genetic data are the basis for any kind of phylogenetic inferences. The ever growing amount of genetic data calls for approaches to dynamically visualize phylogenetic trees that comprise hundreds of thousands of sequences.

The SILVA project provides comprehensive, high-quality datasets of small (16S/18S, SSU) and large (23S/28S, LSU) subunit ribosomal RNA (rRNA) gene sequences for all three domains of life (*Bacteria*, *Archaea*, and *Eukaryota*) [1]. These datasets include large phylogenetic guide trees which need to be interactively visualised for biologist seeking relationships and evolutionary information. For the current release (r128) of the SILVA dataset, the following trees are available:

- SSU Ref NR99 inferred from 645,151 high-quality, full-length SSU rRNA gene sequences, clustered at 99% sequence identity.
- LSU Ref inferred from 154,297 high-quality, full-length LSU rRNA gene sequences.

The SILVA trees include a large amount of metadata about each sequence, which requires several gigabytes of memory footprint, even in compressed form. Currently, browsing such large trees is only possible using desktop applications such as ARB [2], which is only available for Mac OS and Linux. Web-based solutions, like phylo.io [3] or iTOL [4], are not able to effectively handle trees of this dimension. The Open Tree of Life [5] relies on folding and allows the navigation of only one depth level at a time. OneZoom [6] supports large trees but relies on fractals to keep a manageable tree visualization and, hence, does not preserve meaningful branch lengths. In need of an effective, platform independent phylogenetic tree navigation tool, we developed the SILVA Tree Viewer as a web application.

## Implementation

The SILVA Tree Viewer has been developed to provide access to large scale (e.g. 38,585 taxa with 645,151 sequences for the SSU tree) trees in a web browser. The SILVA website hosts the viewer (version 1.1) to provide access to the SSU RefNr99 and LSU Ref phylogenetic guide trees of the SILVA database.

The viewer is based on Web Geographic Information Systems (Web-GIS) technology, in particular, it leverages the PostGIS extension [7] to the PostgreSQL database [8] for the back end, which stores the tree “map”. The Leaflet JavaScript library [9] is used for the front-end, which enables visualization and navigation of the tree as if it was a map. Parsing of the input tree uses the ETE 2 Python library [10]. Additional components have been written in Python to provide features required to satisfy the needs of the biologists. The use of Web-GIS technology enables the viewer to display large scale trees at the cost of not supporting folding.

## Results and discussion

**Fig. 1:**
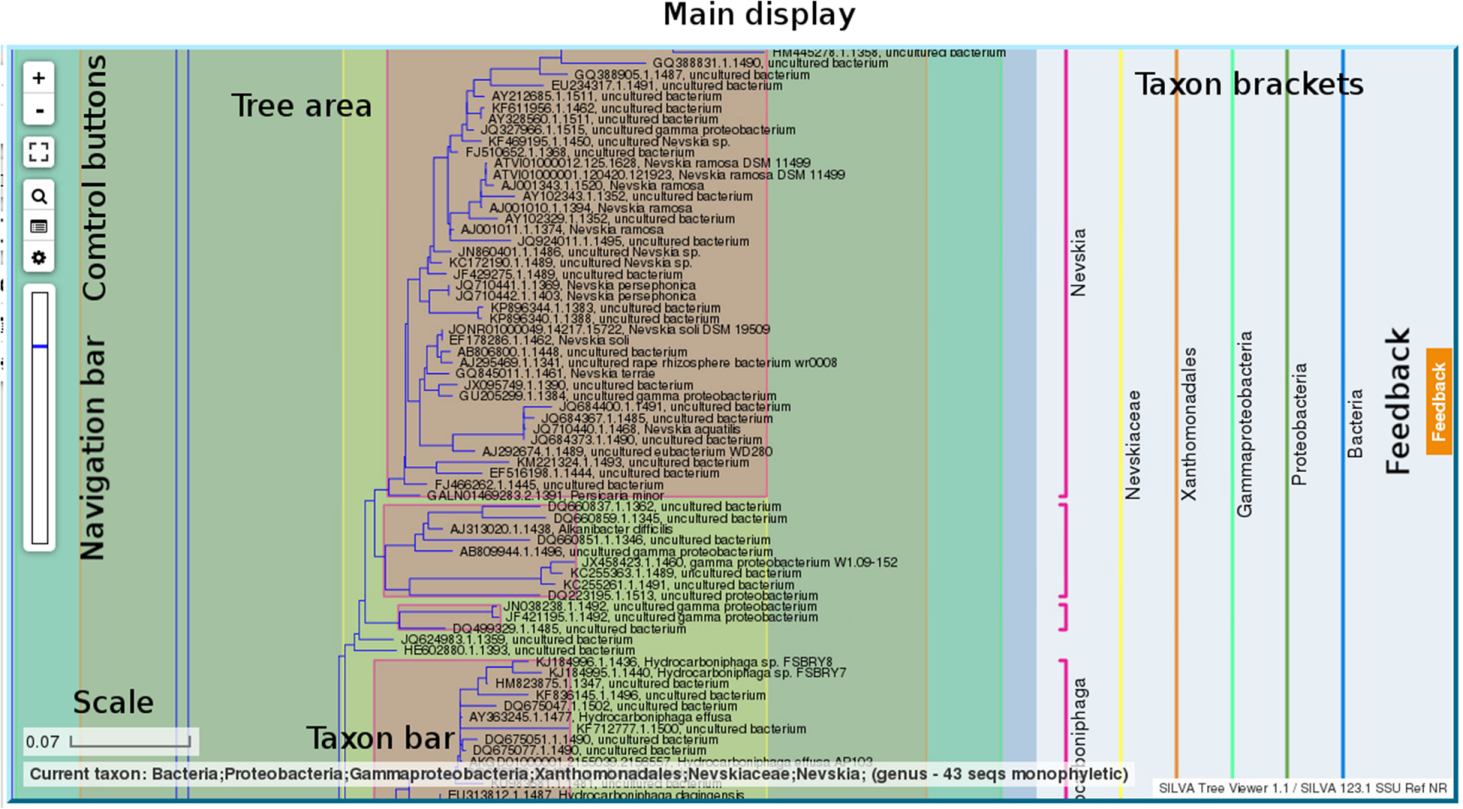
Overview of the SILVA Tree Viewer interface. The following components are available: Main display: shows the SILVA Tree Viewer in the browser or in full-screen mode. Tree area shows the phylogenetic tree; Control buttons to access the main functions; Navigation bar indicates the current view with respect to the whole tree. Taxon bar provides details of a taxon; Taxon brackets: show taxonomic information; Feedback provides access to the feedback system, where support requests - including reports about taxonomic data - can be filed; Scale: provides a reference for interpretation of the horizontal branch length at the current zoom level

The phylogenetic tree is presented to the users with pan and zoom functions similar to those in web mapping technology. A search function, as well as browsing of phyla and major clades, is provided to allow users to quickly lookup sequences and taxa. Additionally, the viewer is equipped with an interactive feedback system which allows reporting of taxonomic problems and asking questions to the tree curators, thus supporting them in improving the quality of the trees. The core components of the SILVA Tree Viewer’s main interface are shown in Fig. 1.

### Tree navigation

**Fig. 2:**
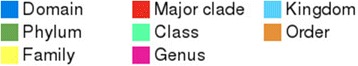
Colour legend of taxonomic rank. Each rank is assigned a different colour to allow clear distinction of taxonomic groups

The tree can be navigated and zoomed in analogy to Google Maps. The different zoom levels allow rapid drill-down navigation from the overview to any area of interest. To further facilitate navigation in large trees, the Navigation bar indicates the current view in relation to the whole tree. The taxonomic groups are shown as coloured rectangles whose colour depend on the taxonomic rank of the group (Fig. 2), enclosing all sequences belonging to them. Further details of any sequence, provided by the SILVA database, can be shown by clicking on its label.

### Taxonomic context

Taxonomic context information is essential for the understanding of the tree. The viewer provides taxonomic context about the currently displayed tree portion in two ways. The Taxon bar, at the bottom of the screen, shows the full path of the taxon under the mouse pointer, along with sequence counts. The Taxonomy Brackets, shown to the right of the visible tree, are vertical square brackets according to the vertical extension of taxonomic groups “crossing” the current view of the tree. They are shown from right to left according to their rank (domain being the rightmost). In case the brackets are too short for the corresponding label to be shown, no label is shown and the taxonomic information remains available via tooltip.

### Search and browse tools

**Fig. 3:**
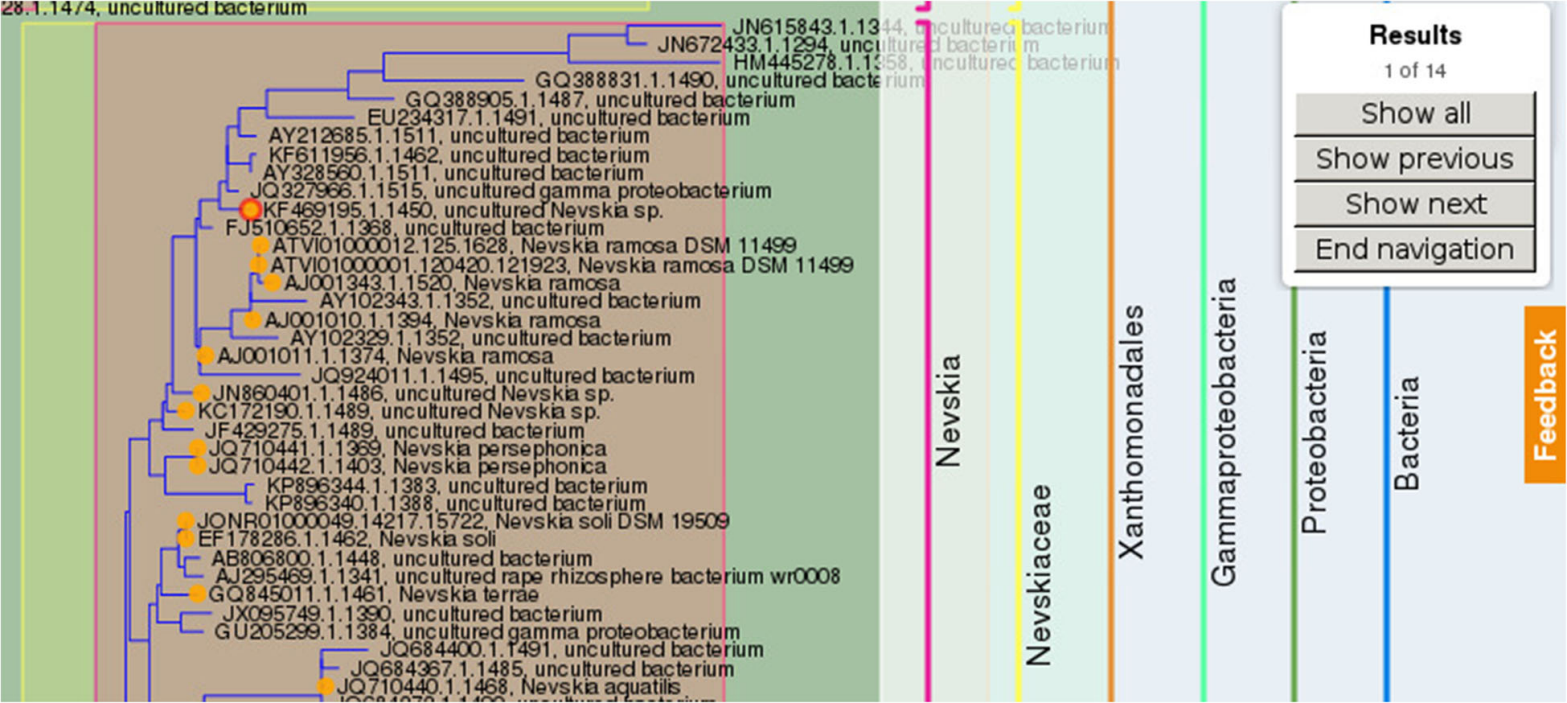
Result set for a search for sequences. All sequences matching the search criteria are marked with an orange dot. The current result is highlighted with a red circle. The top right corner shows the result browser panel, allowing to show all results, navigate to the next/previous result and to leave the results navigator

A search function is provided which allows searching for distinct elements of the tree. The search result is displayed in the viewer (Fig. 3).

### Search for sequences

The user can search for sequences by accession number, returning any sequence whose accession number starts with the query. In this case, the search function can handle start and stop positions, separated by full stops (i.e. the complete accession is composed of <ACCESSION NUMBER > . < START>. < STOP>). Furthermore, a search by full name can be performed, returning all sequences whose species name contains the query string.

### Search for taxa

The user can search for taxonomic groups by taxon name, returning all taxa whose name contains the query. The search by taxonomic path returns all taxa having the query as part of their full taxonomic path is also supported. In this case, since all taxa within a lower rank will match the search condition, only the highest (more generic) rank result will be returned. This search marks taxonomic groups as rectangular features which are loaded to the results navigator for browsing by the user.

### Browse phyla

All taxonomic groups which are direct children of any domain (phyla for *Bacteria* and *Archaea*, kingdom/major clades for *Eukaryota*) are listed for direct browsing. A click on the taxon name loads all taxonomic groups comprising it, zooms the view to the first result and allows navigation through results as for the other search types.

### Feedback system

The feedback system is an important part of the SILVA Tree viewer since the SILVA guide trees are manually curated. The feedback system includes the possibility to report a so-called “data problem”: wrong classifications, errors in the taxonomy and any other problem with the phylogenetic data. This eases the curation process, thus supporting the quality improvement of the dataset in a cooperative effort with the users. Reporting a data problem is a two-step process: first, the data affected has to be selected by picking it interactively on the SILVA Tree Viewer, second, a report form has to be filled to provide details about the problem.

The feedback system can also be used to report on technical problems and to propose improvements to the viewer, as well as for asking general questions to the SILVA team.

### Known limitations and future developments

In its current version (1.1), the SILVA Tree Viewer implements only visualization tools, thus no editing of the trees is supported. Due to technical constraints of the Web-GIS technology there is also no support for group folding, thus the tree is always shown as fully expanded.

Integration with the SILVA search and download system is planned to allow extended search functionality and the direct download of sequence data. The integrated feedback system will provide further guidance for the SILVA team in providing new features, taking into account user suggestions and recommendations.

## Conclusions

The SILVA Tree Viewer enables users worldwide to navigate the large phylogenetic trees provided by the SILVA database. Hundreds of thousands of sequences can be searched in the tree and browsed in their taxonomic context. An interactive feedback system is provided to collect requests for improving the tool itself and guide data curation.

## Availability and requirements

Project name: SILVA Tree Viewer.

Project home page: https://www.arb-silva.de/treeviewer

Operating system(s): Platform independent.

Programming language: Python, JavaScript.

Other requirements: PostgreSQL including PostGIS, Apache.

Any restrictions to use by non-academics: none.
